# Work-related pesticide poisoning among farmers in two villages of Southern China: a cross-sectional survey

**DOI:** 10.1186/1471-2458-11-429

**Published:** 2011-06-03

**Authors:** Xujun Zhang, Weiyan Zhao, Ruiwei Jing, Krista Wheeler, Gary A Smith, Lorann Stallones, Huiyun Xiang

**Affiliations:** 1 Southeast University Injury Prevention Research Institute, Southeast University, Nanjing, Jiangsu Province, China; 2 Center for Injury Research and Policy, The Research Institute at Nationwide Children's Hospital, Columbus, OH, USA; 3 School of Public Health, Southeast University, Nanjing, Jiangsu, China; 4 The Ohio State University College of Medicine, Columbus, OH, USA; 5 Colorado Injury Control Research Center, Colorado State University, Fort Collins, CO, USA

**Keywords:** China, pesticide poisoning, farmers, occupational poisoning

## Abstract

**Background:**

Pesticide poisoning is an important health problem among Chinese farm workers, but there is a paucity of pesticide poisoning data from China. Using the WHO standard case definition of a possible acute pesticide poisoning, we investigated the prevalence and risk factors of acute work-related pesticide poisoning among farmers in Southern China.

**Methods:**

A stratified sample of 910 pesticide applicators from two villages in southern China participated in face-to-face interviews. Respondents who self-reported having two or more of a list of sixty-six symptoms within 24 hours after pesticide application were categorized as having suffered acute pesticide poisoning. The association between the composite behavioral risk score and pesticide poisoning were assessed in a multivariate logistic model.

**Results:**

A total of 80 (8.8%) pesticide applicators reported an acute work-related pesticide poisoning. The most frequent symptoms among applicators were dermal (11.6%) and nervous system (10.7%) symptoms. Poisoning was more common among women, farmers in poor areas, and applicators without safety training (all p < 0.001). After controlling for gender, age, education, geographic area and the behavioral risk score, farmers without safety training had an adjusted odds ratio of 3.22 (95% CI: 1.86-5.60). The likelihood of acute pesticide poisoning was also significantly associated with number of exposure risk behaviors. A significant "dose-response" relationship between composite behavioral risk scores calculated from 9 pesticides exposure risk behaviors and the log odds of pesticide poisoning prevalence was seen among these Chinese farmers (R^2 ^= 0.9246).

**Conclusions:**

This study found that 8.8% of Chinese pesticide applicators suffered acute pesticide poisoning and suggests that pesticide safety training, safe application methods, and precautionary behavioral measures could be effective in reducing the risk of pesticide poisoning.

## Background

Work-related pesticide poisoning remains a pressing public health concern worldwide, especially in less-developed countries [1,2]. The World Health Organization (WHO) reported that about one million serious unintentional pesticide poisoning occur each year [2]. A study of agricultural workers in developing regions of Asia estimated that as many as 25 million cases of mild pesticide poisoning occur annually [3,4]. A significant hurdle to the control and prevention of work-related pesticide poisoning is that the scope and magnitude of this issue often remains uncharacterized, particularly in an underserved population such as farmers [4]. Therefore, the WHO recently created a standard case definition matrix to facilitate the identification, management, and control of acute pesticide poisoning around the world [5]. Unlike workers in pesticide manufacturing companies who may receive safety training to reduce exposure, the majority of farmers in China work independently with small plots of farmland. They may apply pesticides using simple backpack style applicators without adequate knowledge of the necessary precautionary measures.

Pesticide poisoning data in China are often reported as incidence data from hospitals or injury centers. The majority are attempted suicides, but also included are unintentional pesticide poisoning among children [6,7]. A large proportion of farmers who suffer mild pesticide poisoning do not seek professional medical care. Although there are policies in China requiring physicians to report pesticide poisonings, many physicians in rural areas may fail to report cases because they lack the time or administrative resources, and because there are no consequences for not reporting.

With the burden of feeding 20% of the world's population on only 7% of the world's arable land, China has a high reliance on pesticides to maintain crop yields and is currently the largest user, producer and exporter of pesticides [8].

Pesticide exposure has been shown to be an important health problem among Chinese farm workers [9]. Between 1997 and 2003, there were 108,372 cases of pesticide poisoning reported by the National Institute of Occupational Health and Poison Control at the Chinese Center for Disease Control and Prevention, but only 25.4% of the cases were work-related [7]. This low percentage suggests possible underreporting of work-related pesticide poisonings in China [6,7,10]. For example, a study in Shandong Province reported 35 suspected cases of work-related pesticide poisoning among 353 pesticide exposed farmers, suggesting the importance of active surveillance among exposed workers [11].

In this study we conducted an epidemiological survey in a rural area designed: 1) to estimate the prevalence of work-related acute pesticide poisoning among Chinese farmers who applied pesticides; 2) to evaluate pesticide application methods and their association with pesticide poisoning; and 3) to investigate the risk factors for work-related pesticide poisoning.

## Methods

### Study Design and Sampling

In this cross-sectional study, farmers in two villages in Jiangsu Province were surveyed about work-related pesticide poisoning symptoms. The study area was designed to include a lower income area (Subei in the north) and a higher income area (Sunan in the south). According to 2008 statistics the annual income per family member was ¥4010 ($580) in the Subei area, and ¥9200 ($1350) in the Sunan area. One village was selected from each area. Eligible participants included any person who came into contact with pesticides while performing agricultural activities in the 12 months preceding the beginning of the study.

Based on an anticipated prevalence of acute pesticide poisonings among Chinese farmers of 10%, a total of 864 pesticide applicators from two villages were needed to achieve a study power of 80% and an alpha of 5%. Using the population census data from the local government office, two villages with a combined estimated total of 1000 adult farmers were chosen for our study.

The questionnaire was developed by a research team at the Southeast University School of Public Health in Nanjing, Jiangsu Province. Six graduate students from the Southeast University School of Public Health were trained as interviewers for this study. The research team at the Southeast University School of Public Health pilot tested the survey questionnaire in a small group of the target population in the study area in June 2009. Specifically, fifteen people from the two villages were interviewed; these fifteen pesticide applicators were re-interviewed in the larger study. Minor changes were made before the survey questionnaire was finalized. With help from village leaders who located the households, introduced the interviewers, and assisted with communication when it was necessary, data were collected by face-to-face interviews in July and August of 2009. Each participant was asked about family and personal characteristics, types of crops, and activities associated with pesticide application, including safety knowledge, application methods, application time, self-protection methods used, and other behavioral risk factors. In order to investigate the prevalence of work-related poisoning, farmers were asked to report symptoms they suffered if they felt ill during or within 24 hours of pesticide exposure.

### Human Participant Protection

The institutional review boards of Southeast University and Colorado State University reviewed and approved the study procedures. Informed consent was obtained from participants in accordance with ethical guidelines.

### Study Variables

#### Work-related Pesticide Poisoning

Sixty-six symptoms were included in the questionnaire. In addition to general symptoms, the list included symptoms specifically related to the skin, eyes, nervous system, respiratory system, gastrointestinal tract, urogenital system, and cardiovascular system. We utilized the WHO case definition matrix for a possible acute pesticide poisoning [5], to guide our definition of acute pesticide poisoning. Respondents who self-reported having had two or more of the listed symptoms within 24 hours after applying pesticide were considered to have suffered acute pesticide poisoning. Attempted suicide by pesticide, and pesticide poisonings during the production, transportation, or marketing of pesticides (any non-agricultural work-related situations) were excluded from our study.

#### Safety knowledge

Farmers were asked whether they had received any pesticide safety instruction through television, tapes, lectures, written information, oral conversation, or other means.

#### Protective Application Methods

Findings from the pilot survey and our informal discussions with farmers suggested that three simple pesticide application methods might be effective in reducing the risk of acute pesticide poisoning: alternate row spraying; backward application;, and spraying downwind. Alternate row spraying is traveling down every other row when spraying. Backward application is walking backwards while applying pesticides. Spraying downwind means the direction of prevailing winds is determined and spraying is done so that the individuals applying pesticides are not exposed by wind blowing pesticides at them during application. Farmers were asked to report whether or not they often used any of the three application methods in the past year.

#### Behavioral Risk Factors and Composite Risk Score

Based on the literature, we asked the respondents about nine risk behaviors that were thought to increase the risk of acute pesticide poisoning. Respondents could answer yes, no, or don't know. The 'don't know' response was not factored into the composite risk score. The questions are listed below:

1. Did you read labels about the pesticides before application? ("no" = 1, "yes" = 0)

2. Did you prepare pesticides without gloves? ("no" = 0, "yes" = 1)

3. Did you use personal protecting equipment/clothing during application? ("no" = 1, "yes" = 0)

4. Did you smoke cigarettes or eat food during application? ("yes" = 1, "no" = 0)

5. Did you wipe sweat with your hand(s) during application? ("yes" = 1, "no" = 0)

6. Was your knapsack leaking during application? ("yes" = 1, "no" = 0)

7. Did you avoid physical contact with liquid pesticides during application? ("no" = 1, "yes" = 0);

8. Did you continue to work when you felt ill from pesticides? ("yes" = 1, "no" = 0)

9. Did you take a bath after pesticide application? ("no" = 1, "yes" = 0).

A composite score was computed by adding the responses together so the final score could range from 0 to 9. A larger score represented a higher number of risk behaviors based on the coding shown above.

In our analysis, we examined the association between each risk behavior and the prevalence of acute pesticide poisoning. The composite score was analyzed by grouping scores as follows: 0-1; 2-3; 4-5; 6-9).

### Statistical Analysis

Data were entered into EpiData 3.0 and analyzed with SAS 9.1 (SAS Institute, Cary, NC). First, we described the sample demographics in the two geographic areas and listed the frequencies of major symptoms reported by pesticide applicators. Second, χ^2 ^analyses were performed to assess the association of work-related acute pesticide poisoning with selected demographic factors, safety knowledge, pesticide application methods, and the nine risk behaviors. Third, the log odds of acute pesticide poisoning prevalence by composite risk score groups was estimated and summarized in a scatter plot. In order to control for the confounding effects of our study variables, univariate and multivariate logistic regression analyses were conducted. In our logistic regression analyses, the outcome variable was acute pesticide poisoning (yes/no), and the independent variables were gender, age, education level, study area (Sunan vs. Subei areas), whether any pesticide safety training was received, and levels of the final composite risk score. Odds ratios (ORs) and adjusted odds ratios (AORs) with 95% confidence intervals (CIs) were derived from the models. A p < 0.05 was considered statistically significant in our study.

## Results

Using local residence registration information, we estimated that there were 1000 pesticide applicators in the two villages. Our face-to-face survey obtained 910 valid questionnaires, yielding an overall response rate of 91.0%. Reasons for non-response included absence from the village when the survey was conducted and incomplete questionnaires.

A total of 510 farmers were from the Subei village, and 400 farmers were from the Sunan village. The Subei village had higher percentages of young (≤ 54 years) and female (53.1%) applicators, while applicators in the Sunan village were more likely to be older (≥55) and male (80.0%). Education level in the two villages was similar. Fifty-eight percent of the total study sample had received, at most, an elementary level education.

As shown in Table 1, the most frequent symptoms suffered by pesticide applicators were dermal (11.6%) and nervous system symptoms (10.7%), followed by gastrointestinal symptoms (4.4%) and general symptoms (4.1%). Symptoms related to the eyes and the cardiovascular systems were rare (0.3% for each).

**Table 1:** List of symptoms of acute pesticide poisoning self-reported by the 910 pesticide applicators *

	Symptoms list	n of responses (%) for all applicators
Skin	Blister, dermatitis, urticaria, hyperhidrosis, pruritus and swelling	106 (11.6)
Nervous system	Dizziness, syncope, headache, numbness and weakness	97 (10.7)
Gastrointestinal tract	Diarrhea, vomiting and nausea	40 (4.4)
General	Unpleasant smell, taste change, fever, poor appetite, muscular pain and thirsty	37 (4.1)
Respiratory system	Chest pain or chest stuffiness, cough, dyspnea/short breath, laryngeal itch and pain	16 (1.8)
Eyes	Visual fuzzy/double image and eye itch	3 (0.3)
Cardiovascular system	Arrhythmia and tachycardia	3 (0.3)

* Applicators reporting symptoms, n = 124. Applicators reporting two or more symptoms, n = 80.

Table 2 presents the prevalence of acute pesticide poisoning by selected demographic factors. Eighty cases of acute pesticide poisoning (8.8%) were identified in our study. Among those reporting acute pesticide poisoning symptoms, 92.5% of applicators reported they were using insecticides before symptoms occurred; other types of pesticides in use included herbicides (2.5%) and bactericides (5%) (data not shown). The prevalence of acute pesticide poisoning was significantly higher in the Subei village than in the Sunan village (12.2% vs. 4.5%, p < 0.001). Compared with male applicators, a significantly higher proportion of female applicators reported acute pesticide poisoning (13.1% vs. 6.1%, p < 0.001). The proportion of applicators who suffered acute pesticide poisoning was lower among older applicators: 24-34 years (15.0%), 65+ years (5.6%); however, the difference was not statistically significant.

**Table 2:** Comparison of pesticide poisoning by selected demographics *

	Total N. of applicators	Persons with pesticide poisoning n (%)	*P *value*
**Total**	910	80 (8.8)	
**Area**			**< 0.001**
Subei	510	62 (12.2)	
Sunan	400	18 (4.5)	
**Gender**			**< 0.001**
Men	559	34 (6.1)	
Women	351	46 (13.1)	
**Age (years)**			0.578
24-34	20	3 (15.0)	
35-44	150	15 (10.0)	
45-54	308	29 (9.4)	
55-64	324	27 (8.3)	
65+	108	6 (5.6)	
**Education**			0.690
Elementary school or less	526	48 (9.1)	
Middle school	340	27 (7.9)	
High school or more	44	5 (11.4)	

* χ^2 ^analysis of the association between poisoning % and pesticide applicators demographics.

**Figure 1:**
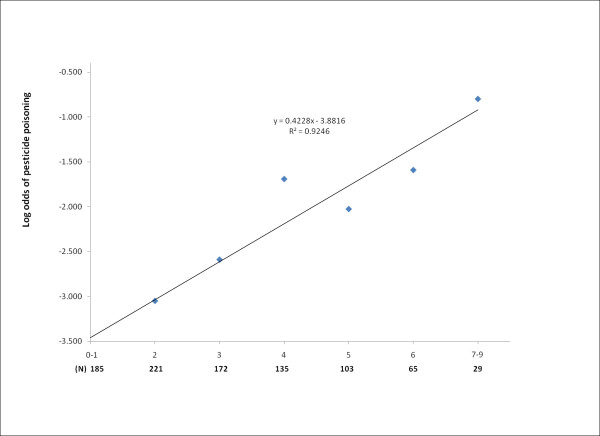
**Relationship between the composite behavioral risk scores and the log odds of acute pesticide poisoning**.

Table 3 describes acute pesticide poisoning prevalence by safety training, three application methods, and nine risk behaviors. The prevalence of acute pesticide poisoning among farmers without any safety training was significantly higher than that among farmers who had received pesticide safety training (12.4% vs. 5.4%, p < 0.001). Farmers who reported risky behaviors (such as not using personal protective appliances, having had a leaky knapsack, not avoiding physical contact with liquid pesticides, or continuing to apply pesticides when feeling ill) had significantly higher prevalence of acute pesticide poisoning than farmers who did not report these behaviors (all p ≤ 0.01). Farmers who did not use protective application methods during pesticide applications reported higher percentages of acute pesticide poisoning than farmers who used protective application methods, although the difference between groups was not statistically significant. When the final composite risk scores were plotted against the log odds of acute pesticide poisoning prevalence, ln(), our results (Figure 1) suggested that there was a significant relationship between the composite behavioral risk scores and the odds of acute pesticide poisoning (R^2 ^= 0.9246).

**Table 3:** Association of pesticide poisoning with safety knowledge, application methods and personal protection behaviors

	Response	n ^†^	Acute Pesticide poisoning (%)	*P *value*
**Safety knowledge**				
Received instruction	Yes	479	5.4	**< 0.001**
	No	429	12.4	
**Application methods**				
Alternate row spraying	Yes	137	5.8	0.181
	No	769	9.4	
Backward application	Yes	492	7.5	0.130
	No	414	10.4	
Down-wind application	Yes	653	8.0	0.140
	No	253	11.1	
**Behaviors**				
Read label	Yes	533	7.9	0.243
	No	376	10.1	
Prepare pesticides without gloves	Yes	798	8.8	0.673
	No	106	7.6	
Use protective appliances	Yes	709	7.5	**0.010**
	No	195	13.3	
Smoking/eating when applying	Yes	53	15.1	0.098
	No	853	8.4	
Wipe sweat with hand(s)	Yes	336	12.2	**0.006**
	No	570	6.8	
Equipment leakage	Yes	378	13.2	**< 0.001**
	No	528	5.7	
Avoid body pollution by pesticide	Yes	384	3.4	**< 0.001**
	No	521	12.7	
Work when sick	Yes	241	17.0	**< 0.001**
	No	647	5.9	
Bath after work	Yes	846	8.9	0.280**
	No	12	0.0	

† n is less than 910 because of missing data.

* χ^2 ^statistical test of difference in pesticide poisoning %

** Fisher's exact test

Results from the univariate and multivariate logistic regression models are reported in Table 4. After controlling for the covariates (age, education, area, safety knowledge and behavioral risk score), acute pesticide poisoning was 70% more likely to occur among female than among male applicators (AOR = 1.70, 95% CI: 0.99-2.91), but the difference was only marginally significant (p = 0.055). Acute pesticide poisoning was more likely to occur among applicators in the less prosperous Subei village than in the Sunan village (AOR = 2.28, 95% CI: 1.24-4.21). Applicators who did not receive any type of pesticide safety training had AOR of acute pesticide poisoning over 3 times that seen in those who had received safety training (*P *< 0.013). Compared with applicators who had composite risk scores of 0-1, applicators with risk scores of 6-9 had considerably higher risks of pesticide poisoning (AOR = 6.49, 95% CI = 2.17-19.46 ), and applicators with risk scores of 4-5 were nearly 3.7 times likely to suffer from pesticide poisoning (AOR = 3.67, 95% CI: 1.32-10.18). A dose-response relationship was observed between composite risk scores and prevalence of acute pesticide poisoning in the final logistic model.

**Table 4:** Logistic regression models of pesticide poisoning

	Univariate analysis		Multivariate analysis	
		
	OR (95% CI)*	*P *value	AOR (95%CI)**	*P *value
**Gender**				
Woman	2.33 (1.46-3.71)	**< 0.001**	1.70 (0.99-2.91)	0.055
Man	-		-	
**Age group (years)**				
24-34	3.00 (0.68-13.15)	0.145	2.76 (0.56-13.58)	0.211
35-44	1.89 (0.71-5.04)	0.204	1.56 (0.54-4.52)	0.414
45-54	3.12 (1.08-9.02)	0.219	1.75 (0.65-4.68)	0.267
55-64	1.54 (0.62-3.85)	0.350	1.99 (0.75-5.26)	0.166
65+	-		-	
**Education**				
Elementary school or less	1.16 (0.71-1.91)	0.545	0.78 (0.44-1.38)	0.384
Middle school	-		-	
High school or more	1.49 (0.54-4.08)	0.442	1.53 (0.53-4.39)	0.430
**Area**				
Sunan	-		-	
Subei	2.94 (1.71-5.058)	**< 0.001**	2.28 (1.24-4.21)	**0.008**
**Safety knowledge**				
Yes	-		-	
No	2.46 (1.51-4.00)	**< 0.001**	3.22 (1.86-5.60)	**0.013**
**Behavioral risk score**				
Level 1 (score 0-1)	-		-	
Level 2 (score 2-3)	2.13 (0.80-5.73)	0.132	1.78 (0.65-4.91)	0.265
Level 3 (score 4-5)	5.80 (2.21-15.16)	**< 0.001**	3.67 (1.32-10.18)	**0.013**
Level 4 (score 6-9)	9.73 (3.52-26.89)	**< 0.001**	6.49 (2.17-19.46)	**< 0.001**

* OR (95%CI), odds ratio of pesticide poisoning with 95% confidence interval

** AOR (95%CI), adjusted odds ratio of pesticide poisoning with 95% confidence interval

## Discussion

Our study indicates that 8.8% of Chinese farmers in the study areas who applied pesticides in the past year suffered work-related acute pesticide poisoning. Our study suggests that acute pesticide poisoning is significantly associated with factors such as geographic area, and whether the farmers received pesticide safety training. More importantly in this cohort, we identified the importance of certain behaviors in increasing the risk of acute pesticides poisonings. Our findings indicate how important effective education programs regarding pesticide safety could be in preventing pesticide poisoning among Chinese farmers.

While pesticide poisoning is common in less developed countries, this seems to be especially true in China. The 8.8% prevalence found here in Jiangsu province is close to what was reported in a previous study among farmers by Lin, et al. (9.9% acute pesticide poisonings, 35/353) in Shandong Province (Jiangsu shares Shandong's southern border) [11]. These prevalence rates are higher than those reported in other Asian developing countries (0.3% in Indonesia, 7.1% in Sri Lanka, and 7.3% in Malaysia) [12], in Central America [13], and in developed countries such as the United States [14]. However, it is important to note when making international comparisons that the definition of acute pesticide poisoning, the farming practices, and study designs vary across the studies so that the absolute risk differences based on prevalence rates cannot be determined.

Pesticide poisoning prevalence among female applicators was nearly two-fold greater than that of male applicators, but in multivariate modeling, safety knowledge and the risk behavior scores were more important variables than gender. Gender differences in acute pesticide poisoning have also been reported among agricultural workers in the United States [14]. Female vulnerability to pesticides may have physiological [15], behavioral and socioeconomic roots. Females may have a significantly lower percentage of self-protective behaviors during pesticide application than males [16] and gender-related working conditions might also aggravate work-related exposure to agricultural pesticides among female applicators [17,18].

Geographic region was an important factor for acute pesticide poisoning in our study. Subei applicators had a markedly higher risk of pesticide poisoning than those from the Sunan village. This difference may be due to the use of different types of pesticides based on the crops or differences in the availability of specific compounds. Since the study did not collect specific pesticide information it is not possible to determine if that accounted for a higher prevalence of acute pesticide poisoning in the lower income area.

Safety knowledge and protective behaviors were significantly associated with a lower prevalence of work-related pesticide poisoning. Previous studies have suggested that pesticide safety education among farmers could raise awareness of both pesticide exposure risk [19] and the adverse health consequences associated with acute pesticide poisoning [20]. Improvements in pesticide safety knowledge using different delivery modes may lead to some improvement in protective practices [21] and increase the use of personal protective equipment [22]. In environments with low literacy as in our study area, training programs using oral presentations and storytelling could provide a basic safety education and help farmers to understand pesticides manufacturers' complex labeling information [23]. However, reducing the risk of pesticide poisoning may require behavioral changes. We found a significant relationship between the composite behavioral risk scores and the odds of acute pesticide poisoning, with more risky behaviors leading to a higher likelihood of acute pesticide poisoning. Increased risk of exposure may also apply to the applicators' family members and children, if pesticide residues on applicators' bodies or clothes are carried into the home [24].

The strength of this study was the use of the WHO case classification matrix to identify acute pesticide poisoning cases. Based on this case definition matrix, laboratory confirmation is not required to meet a standard definition of possible acute pesticide poisoning. Use of this standard definition makes it possible to compare our results with other studies using the same WHO case classification. An additional strength of the study is the innovative use of a composite pesticide exposure risk score.

Several limitations of our study should be noted. Our research team did not directly observe the behavior of the participants. Instead, information on acute pesticide poisoning and related factors was collected through a cross-sectional survey. Recall bias may lead to an inaccurate estimation of the prevalence of acute pesticide poisoning and the examined behaviors. A second limitation is that this study did not collect comprehensive information about each applicator's pesticide application intensity and duration. Instead, this small field study of a trainee of the USA-China Agricultural Injury Research Training Programs focused on pesticide safety education and key pesticide exposure risk behaviors that could be changed by education programs and other interventions.

## Conclusions

At present, pesticides remain an integral part of agricultural activities in many parts of the world. There are increasing efforts to develop safer alternatives to pesticides, but economic realities may influence whether these options are considered viable ones for many less developed nations. Findings from this study suggest that pesticide safety education and use of protective application methods could be effective in reducing the risk of acute pesticide poisoning. A warranted next step is the development of educational programs to teach Chinese farmers to practice precautionary measures when working with pesticides.

## Competing interests

The authors declare that they have no competing interests.

## Authors' contributions

XZ, LS, and HX conceived and designed the project. XZ and RJ were responsible for data collection. XZ, WZ, KW, GS, LS, and HX were involved in the data analysis, drafting the article, its critical review. All authors read and approved the final manuscript.

## Pre-publication history

The pre-publication history for this paper can be accessed here:

http://www.biomedcentral.com/1471-2458/11/429/prepub
